# Aberrant HSP90 Expression in Lymphocytes and HSP90 Response to Anti-PD-1 Therapy in Lymphoma Patients

**DOI:** 10.3389/fimmu.2022.893137

**Published:** 2022-04-28

**Authors:** Zarema Albakova, Yana Mangasarova, Akhmet Albakov, Elena Nikulina, Sergey Kravchenko, Alexander Sapozhnikov

**Affiliations:** ^1^Department of Immunology, Lomonosov Moscow State University, Moscow, Russia; ^2^National Medical Research Center for Hematology, Moscow, Russia; ^3^Department of Innovation, Chokan, Almaty, Kazakhstan; ^4^Department of Immunology, Shemyakin and Ovchinnikov Institute of Bioorganic Chemistry Russian Academy of Sciences (RAS), Moscow, Russia

**Keywords:** HSP90, lymphocytes, cancer immunotherapy, extracellular HSP90, PD-1 blockade, Hodgkin lymphoma, Non-Hodgkin lymphoma

## Abstract

HSP90 family of molecular chaperones has been shown to be implicated in various stages of tumor growth and development. Recent studies have highlighted the role of extracellular HSP90 in tumor immunology, however, the role that HSP90 plays in the regulation of immune responses and the impact of cancer immunotherapy, including immune checkpoint blockade, on HSP90 is still unclear. Here we assessed the surface and intracellular expression of constitutive cytosolic HSP90β isoform, mitochondrial HSP90 homolog TRAP1 and co-chaperone STIP1/HOP in T, NK, B and NKT cells derived from peripheral blood and bone marrow samples of patients with Hodgkin and B-cell Non-Hodgkin lymphomas. HSP90β and STIP1 were overexpressed in B lymphocytes, while TRAP1 expression was decreased in T, B, NK and NKT cells of lymphoma patients. HSP90 overexpression in B cells was not associated with malignant B cell clones, since no clonotypic B cells were detected by immunoglobulin heavy chain (IgH) gene rearrangements. PD-1 blockade was found to differently affect the intracellular and surface HSP90 in T, B, NK and NKT cells in patients with relapsed or refractory classical Hodgkin lymphoma. Modulating HSP90 was found to affect the NK cell degranulation response and IFNγ production in lymphoma patients. These findings provide the rationale to further explore HSP90 homologs for improving patient response to cancer immunotherapy.

## Introduction

HSP90 family of molecular chaperones plays crucial in protein folding, degradation and maturation of client proteins (1, 2). HSP90 family is composed of four homologs, such as stress-inducible HSP90α, constitutive HSP90β, tumor necrosis factor receptor-associated protein 1 (TRAP1) and glucose-regulated protein 94 (GRP94) (1, 3, 4). HSP90α and HSP90β isoforms primarily reside in cytosol, TRAP1 in mitochondria and GRP94 in endoplasmic reticulum (ER), where HSP90 isoforms act in a variety of cellular processes, including unfolded protein response, mitochondrial metabolism, lipid metabolism, autophagy and apoptosis (4). Cytosolic HSP90s work in collaboration with co-chaperones, including HSP70-HSP90 organizing protein (HOP/STIP1), protein phosphatase 5, cyclophilin 40, FK506-binding protein, activator of HSP90 ATPase homolog 1 (Aha1), p23 and cell division cycle 37 (Cdc37) (5–7). Under various stress conditions, HSP90 homologs may translocate from their primary location and can be released into the extracellular milieu (8–10). In the context of cancer, HSP90 homologs have been shown to be implicated in the regulation of epithelial-mesenchymal transition, metastasis, cancer cell stemness, invasion, apoptosis resistance and tumor immunity [reviewed in (4)].

Lymphoma is a heterogeneous group of tumors divided into two main types, such as Hodgkin lymphoma (HL) and Non-Hodgkin Lymphoma (NHL) (11, 12). Classical HL (cHL) is the most common subtype of HL, which is characterized by the presence of malignant Hodgkin and Reed-Sternberg (HRS) cells (13, 14). Even though HRS cells are germinal cell –derived B cells, they rarely express classical B cell markers (11). NHL lymphoma primarily consists of B-cell lymphomas (BCL) while other NHL subtypes include T- and NK- cell lymphomas (15). HL patients are usually treated with chemotherapy and radiotherapy while NHL patients are treated with chemotherapy combined with anti-CD20 (11, 12, 16). Even though the response rate is high, relapses occur in substantial number of lymphoma patients. Relapsed or refractory (r/r) cHL patients are treated with high-dose chemotherapy followed by an autologous hematopoetic stem cell transplantation (ASCT) (11). r/r cHL can also be treated with Nivolumab, an inhibitor of immune checkpoint programmed death-1 (PD-1) (11, 17). Even though cancer immunotherapy showed encouraging results in r/r patients, still some patients do not benefit from it, suggesting that it is critical to identify patients who will likely respond to the therapy.

In our previous study, we have used machine learning to show that HSP90β and TRAP1 are aberrantly expressed in the urine of cancer patients and that the HSP90β, TRAP1 and co-chaperones can be used to identify cancer patients (18). Since lymphoma originates from lymphocytes we sought to analyze the expression of HSP90β, TRAP1 and STIP1 co-chaperone in peripheral blood and bone marrow lymphocytes of patients with Hodgkin and Non-Hodgkin lymphomas. We show that B lymphocytes have the highest expression of HSP90β and STIP1 in lymphoma patients. We also show that PD-1 blockade differentially affects intracellular and surface HSP90s content in lymphocytes of r/r cHL patients. Since HSP90s may modulate immune responses, altering HSP90 expression and localization may further affect functional activity of immune cells. In this regard, we found that HSP90 downregulation impairs NK cell degranulation response and IFNγ production. To the best of our knowledge, this is the first study to assess the expression of HSP90β, TRAP1 and STIP1 in peripheral blood and bone marrow lymphocytes and the role of anti-PD-1 immunotherapy on HSP90 expression in cancer patients.

## Materials and Methods

### Patient Samples

Peripheral blood (PB) and bone marrow (BM) samples were collected from B-NHL (n=5) and cHL (n=3) patients and healthy individuals (n=4). B-NHL group consisted of patients with diffuse large B-cell lymphoma (DLBCL, n=3) and primary mediastinal large B-cell lymphoma (PMBCL, n=2) while cHL group consisted of patients with nodular sclerosis HL (NSHL, n=3). HL and B-NHL patients included in the study were newly diagnosed patients with no previous history of treatment, unless otherwise specified. Samples were also obtained from relapsed or refractory cHL patients (n=3) receiving Nivolumab (Opdivo, Bristol-Myers Squibb) prior to the therapy and after 24 hours post-Nivolumab treatment. All patients were Epstein-Barr virus (EBV)- negative to exclude EBV-associated lymphomas. The median age of patients was 42 years old. Peripheral blood mononuclear cells (PBMCs) and bone marrow mononuclear cells (BM MNCs) were isolated using Ficoll-Paque density gradient centrifugation. The study was approved by the Research Ethics Committee of the Federal State Budgetary Institution ‘National Medical Research Center for Hematology’ of the Ministry of Health of the Russian Federation. All subjects had provided written informed consent in accordance with the Declaration of Helsinki.

### Antibodies and Flow Cytometry

Cells were stained with fluorescently conjugated anti-human antibodies: APC/Cy7 anti-CD3 (HIT3a), APC anti-CD19 (HIB19), Pacific Blue anti-CD3 (HIT3a), FITC anti-human IFNγ (4S.B3) (all Sony Biotechnology), PE-Vio 770 anti-CD56 (REA196), FITC anti-Granzyme B (REA226), APC/Cy7 anti-CD107a (LAMP-1) (H4A3) (all Miltenyi Biotec).

### HSP90β, TRAP1 and STIP1 Surface and Intracellular Staining

Cells were stained with anti-human TRAP1-RPE (3H4-2H6, Sigma-Aldrich), primary antibody against HSP90β (EPR16621), STIP1 (EPR6605) and the secondary antibody goat anti-rabbit IgG H&L PE preadsorbed (all Abcam). Mouse IgG1-PE (Invitrogen) and PE-rabbit IgG (Abcam) were used as isotype controls. FcR blocking reagent (Miltenyi Biotec) was used to block non-specific binding. For intracellular staining, cells were fixed and permeabilized with Cytofix/Cytoperm (BD Biosciences) and stained with antibodies for intracellular proteins. For surface and intracellular staining, dead cells were excluded from gating with the use of Sytox Blue dead stain and Fixable Viability Dye eFluor 506 (Invitrogen), respectively.

### IgH Gene Rearrangement Detection

B-cell clonality (IgH gene rearrangements) was assessed using fragment analysis for V-D-J rearrangements of IgH (FR1, FR2, FR3), as previously described (19). The reaction mixture included 100–200 ng of DNA. PCR conditions: initial denaturation at 95°C (5 min), 35 cycles of PCR at 92°C (35s), 60°C (35s) and 72°C (35s) and final elongation at 72°C (10 min). PCR was performed on an automatic thermal cycler DNA Engine (BioRad, Hercules, USA). The ABI PRISM 3130 Genetic Analyzer (Applied Biosystems, USA) was used for fragment analysis of PCR products. Results were visualized using the GeneMapper v. 4.0 (Applied Biosystems, USA).

### NK Cell Stimulation, HSP90 Inhibition, CD107a/Granzyme B and IFNγ Analysis

NK cells were stimulated as previously described (20). Briefly, PBMCs and BM MNCs were incubated in RPMI 1640 with L-glutamine (Capricorn Scientific), supplemented with 10% Fetal Bovine Serum (FBS, Capricorn Scientific) and penicillin/streptomycin (Capricorn Scientific) with HSP90 inhibitor - geldanamycin (GA, 0.1µM) (Abcam) or DMSO in the presence or absence of the recombinant human (rh) IL-2 (100 IU/ml) (Miltenyi Biotec) and rhIL-15 (10 ng/ml) (Miltenyi Biotec) overnight at 37°C 5% CO_2_ prior to the addition of APC-Cy7 anti-CD107a (Miltenyi Biotec). Cells then were stimulated with anti-NKp46/anti-CD2 (human NK cell activation/expansion kit, Miltenyi Biotec) for 5 hours at 37°C, according to the manufacturer instruction. The incubation was done in complete RPMI medium, supplemented with Brefeldin A (Sony Biotechnology) at a final dilution of 1/1000. Cells were then stained for surface markers and intracellular Granzyme B (Miltenyi Biotec) or IFNγ (Sony Biotechnology) and analyzed by flow cytometry.

### Statistics

All statistical analyses were performed using GraphPad Prism 9. Results are expressed as mean ± standard error of the mean (SEM). In accordance with the data distribution, parametric tests including two-sample t-test and ANOVA and non-parametric methods including Mann Whitney test were employed for the data analysis. P values < 0.05 were considered to be statistically significant.

## Results

### Immune Subpopulations in PBMCs and BM MNCs in HL and NHL Patients

**Figure 1:**
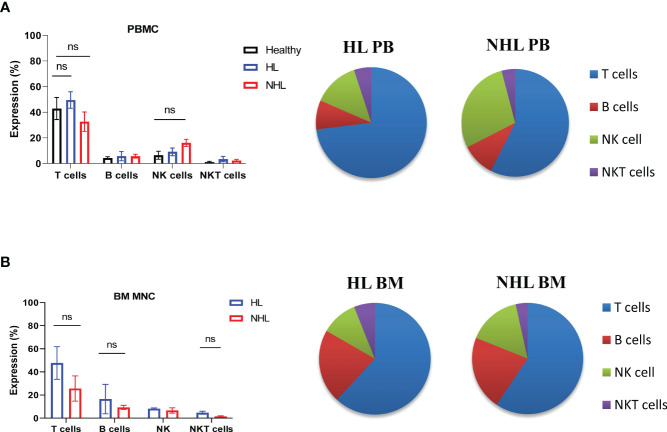
Frequency of lymphocytes in peripheral blood and bone marrow samples derived from patients with HL and NHL patients. The percentage of T cells (CD3^+^ CD56^-^), B cells (CD3^-^CD19+), NK cells (CD3^-^, CD56^+^) and NKT cells (CD3^+^CD56^+^) in peripheral blood **(A)** derived from HL, NHL patients and healthy controls and bone marrow samples **(B)** derived from HL and NHL patients. Graphs show mean ± SEM. ns, not significant. PB, peripheral blood; BM, bone marrow.

Patients with newly diagnosed HL and B-NHL lymphoma had abnormal frequency of lymphocytes in peripheral blood compared to healthy controls (**Figure 1A**). HL and NHL patients differed by the frequency of immune population in PBMCs and BM MNCs (**Figure 1**). HL patients had higher frequency of T cells (CD3^+^CD56^-^) and NKT (CD3^+^CD56^+^) cells in peripheral blood and bone marrow compared to NHL patients. NHL patients had higher frequency of peripheral blood NK cells compared to HL patients, however, the difference was not statistically significant (p>0.05) (**Figure 1A**). Increased frequency of B cells (CD19^+^CD3^-^) and decreased frequency of NK cells (CD56^+^CD3^-^) were observed in bone marrow compared to peripheral blood in HL and NHL lymphoma (**Figure 1**).

### Intracellular and Surface HSP90β, TRAP1 and STIP1 Expression in PB- and BM-Derived Lymphocytes in Lymphoma Patients

**Figure 2:**
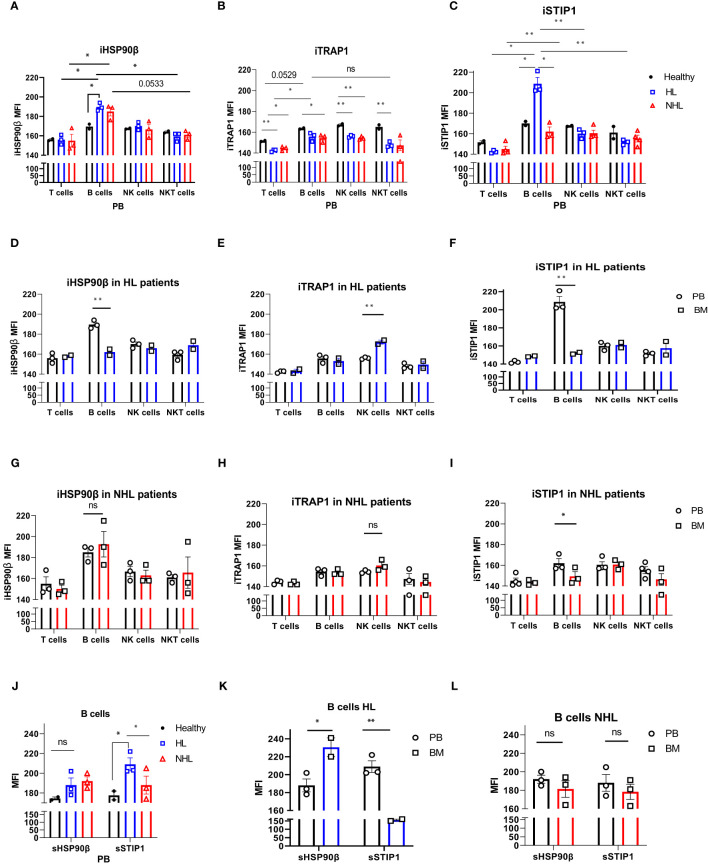
Intracellular and surface HSP90β, TRAP1 and STIP1 expression in PB- and BM- derived lymphocytes of lymphoma patients. The mean level of intracellular expression of HSP90β (iHSP90β) **(A)**, iTRAP1 **(B)** and iSTIP1 **(C)** (as mean fluorescence intensity; MFI) in lymphocytes derived from PB of HL (n=3), NHL (n=3) patients and healthy controls (n=2). The expression of iHSP90β **(D)**, iTRAP1 **(E)** and iSTIP1 **(F)** in lymphocytes derived from BM of HL (n=2) and iHSP90β **(G)**, iTRAP1 **(H)** and iSTIP1 **(I)** in NHL (n= 3) patients. **(J–L)** Surface HSP90β and STIP1 expression in B lymphocytes derived from PB and BM samples of lymphoma patients. **(J)** The expression of sHSP90β and sSTIP1 in B cells derived from PB of HL (n=3), NHL patients (n=3) and healthy controls (n=2). **(K)** The expression of sHSP90β and sSTIP1 in B cells derived from PB and BM of HL (n=2). **(L)** The expression of sHSP90β and sSTIP1 in B cells derived from PB and BM of NHL (n=3) patients. Graphs show mean ± SEM. ns, not significant, *p<0.05, **p< 0.01.

The intracellular expression of HSP90β (iHSP90β), iTRAP1 and iSTIP1 varied in peripheral blood lymphocytes of NHL and HL patients. B cells showed significantly higher iHSP90β expression compared to other peripheral blood lymphocytes (T, NK and NKT cells) in lymphoma patients (**Figure 2A**). iHSP90β and iSTIP1 were significantly overexpressed in peripheral B cells of HL patients compared to healthy controls (**Figures 2A, C**). Bone marrow B lymphocytes showed significantly lower iHSP90β and iSTIP1 expression compared to peripheral B cells in HL patients (**Figures 2D, F**). By contrast, iHSP90β and iTRAP1 expression was comparable in BM- and PB- derived B cells of NHL patients (**Figures 2G, H**). Similar to HL patients, PB-derived B cells showed increased expression of iSTIP1 in NHL patients (**Figure 2I**). iTRAP1 was significantly decreased in peripheral blood lymphocytes in lymphoma patients compared to healthy controls (**Figure 2B**). Notably, HL patients showed higher iTRAP1 expression in bone marrow NK cells compared to peripheral blood NK cells (**Figure 2E**). sSTIP1 was significantly increased on the surface of B cells in peripheral blood of patients with HL lymphoma compared to healthy controls and NHL patients (**Figure 2J**). sHSP90β was significantly increased in bone marrow-derived B cells compared to the peripheral blood B cells of HL patients while sSTIP1 was significantly increased in peripheral blood B cells compared to bone marrow B cells in HL patients (**Figure 2K**). By contrast, sHSP90β and sSTIP1 were decreased in BM-derived B cells compared to peripheral blood B cells in NHL patients, however the difference was not statistically significant (p>0.05) (**Figure 2L**). These data suggest that surface and intracellular expression of HSP90β vary between peripheral blood and bone –marrow B cells and that the HL and NHL lymphomas differ by the expression of intracellular and surface HSP90β and STIP1 in B lymphocytes.

### B-Cell Clonality Analysis in HL and NHL Patients

**Figure 3:**
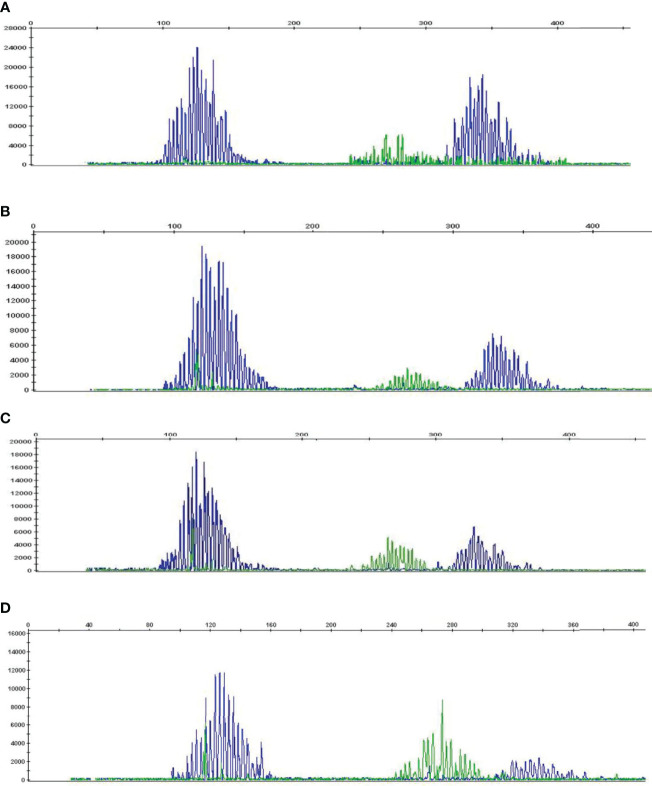
B-cell clonality analysis (IgH gene rearrangements) in PB and BM of HL and NHL patients. Representative graphs showing a Gaussian distribution of multiple peaks in **(A)** peripheral blood and **(B)** bone marrow in NHL patient and **(C)** peripheral blood and **(D)** bone marrow in HL patient.

To determine whether high HSP90 expression in B cells is associated with malignant B cell clones, we performed B-cell clonality analysis (IgH gene rearrangements) using PB and BM samples from HL and NHL patients (**Figure 3** and **Supplementary Figure 3**). No malignant B cell clones were detected in PB and BM of HL and NHL patients (**Figures 3A**–**D** and **Supplementary Figure 3**), suggesting that high HSP90 expression in B cells may not be associated with malignant B cell phenotype.

### Anti-PD-1 Treatment Affects HSP90s Expression in Lymphocytes of r/r cHL Patients

**Figure 4:**
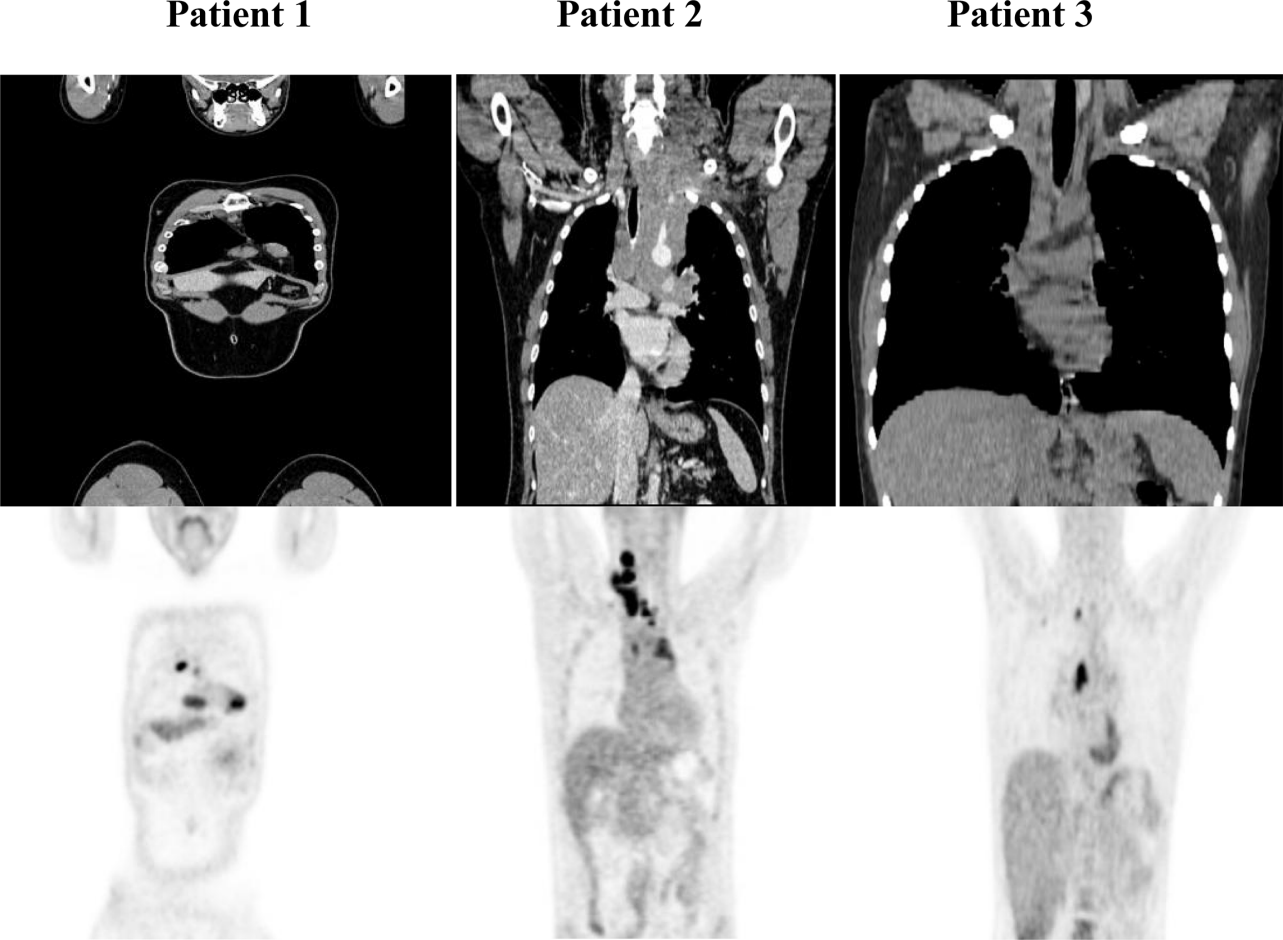
PET/CT of r/r cHL patients prior to the initiation of anti-PD-1 treatment. Patient 1 presented with conglomerate lymph node masses at the anterosuperior mediastinum with the size of 37x21 mm, SUV 6.4 and the paragastric conglomerate with the size of 45x42 mm, SUV 7.4. Patient 2 presented with cervical lymph nodes with the size of 6.6mm, SUV 4.7 and with conglomerate lymph node mass at the anterosuperior mediastinum with the size of 74x42 mm, SUV 7.7. Patient 3 presented with conglomerate lymph node mass at the anterosuperior mediastinum with the size of 43x32 mm, SUV 8.2. SUV, standardized uptake value.

**Figure 5:**
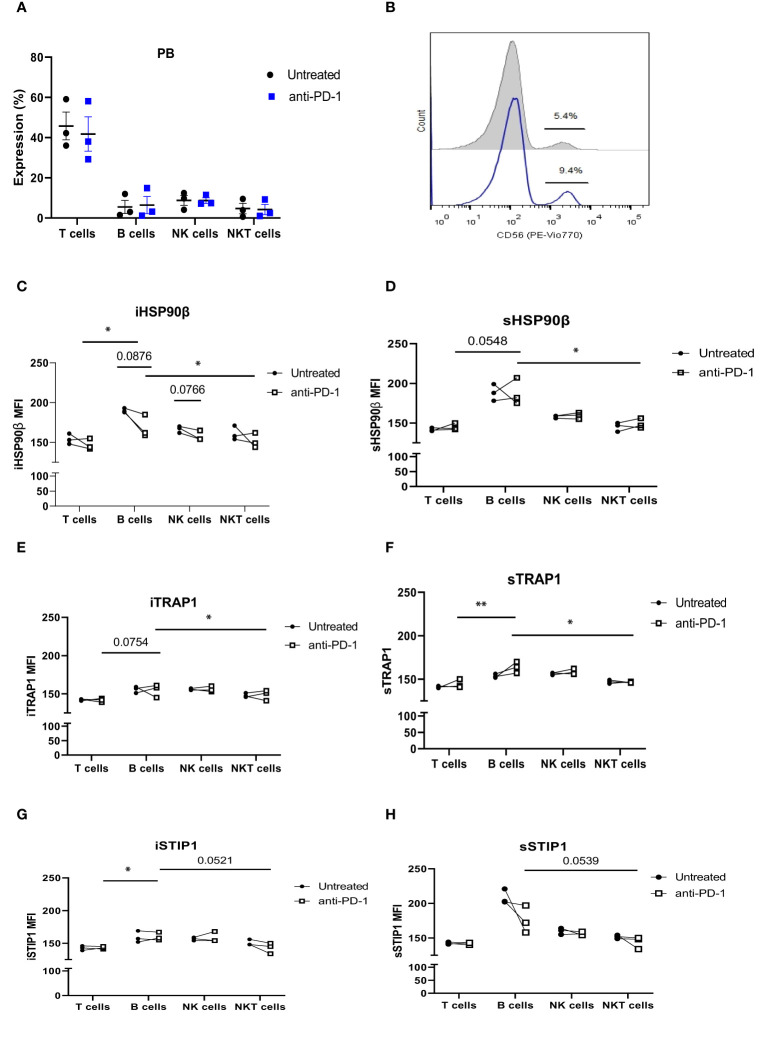
The effect of anti-PD-1 treatment on HSP90 expression in peripheral blood lymphocytes in r/r cHL patients. **(A)** Frequencies of T, B, NK and NKT cells following 24hr treatment with Nivolumab in r/r cHL patients (n=3). **(B)** Representative histogram showing increase in NK cell frequency after 24hr treatment with Nivolumab. iHSP90β expression **(C)** and sHSP90β **(D)**, iTRAP1 **(E)** and sTRAP1 **(F)**, iSTIP1 **(G)** and sSTIP1 **(H)** in peripheral blood lymphocytes of r/r cHL patients at 24 hrs treatment with Nivolumab (n=3). ns, not significant, *p<0.05, **p< 0.01.

Patients with refractory or relapsed cHL undergoing Nivolumab treatment were presented with conglomerate lymph node masses at diagnosis (**Figure 4**). We assessed the effect of anti-PD-1 therapy on the frequency of immune cells and the expression of HSP90β, TRAP1 and STIP1 in T, B, NK and NKT cells prior to and 24 hours after the treatment of patients with r/r cHL. Blocking PD-1 affected the frequency of immune cell population in the peripheral blood at 24 hours of the treatment (**Figure 5A**). The median percentage of T cells was decreased after 24 hours of anti-PD-1 treatment (**Figure 5A**). One patient showed increased frequency of peripheral blood NK cells (**Figure 5B**). Increased frequency of NK cells after anti-PD-1 therapy has been also shown previously in cancer patients (21).

**Figure 6:**
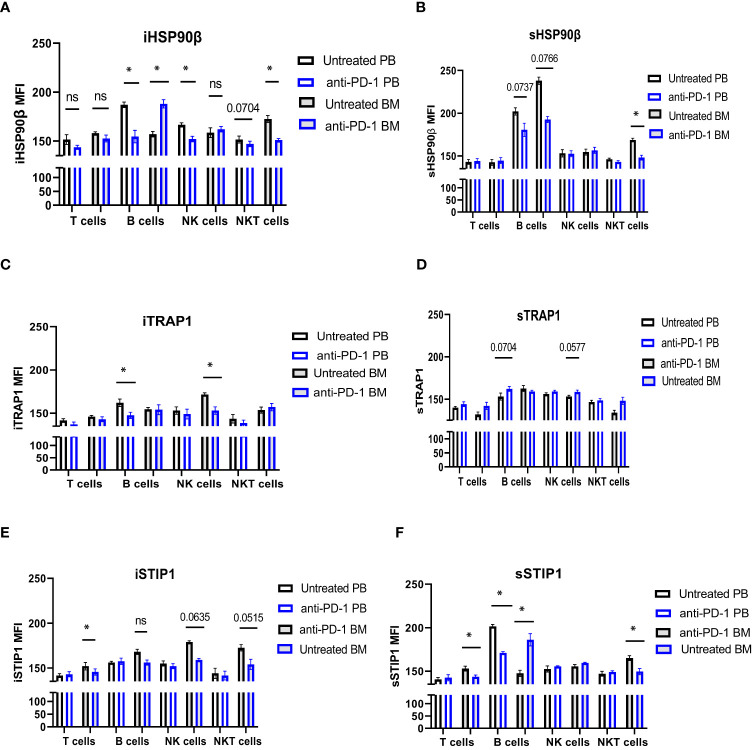
The effect of anti-PD-1 on the HSP90 expression in PB and BM lymphocytes in r/r cHL patient. Intracellular **(A, C, E)** and surface **(B, D, F)** expression of HSP90β **(A, B)**, TRAP1 **(C, D)** and STIP1 **(E, F)** in PB and BM lymphocytes in r/r cHL patient (n=1). Graphs show mean ± SD. ns, not significant, *p<0.05. PB, peripheral blood; BM, bone marrow.

iHSP90β decreased in T, B, NK and NKT cells. Two patients showed increased HSP90β expression on the surface of peripheral blood B cells (**Figures 5C, D**). PD-1 blockade did not affect iTRAP1 expression, but increased sTRAP1 (**Figures 5E, F**). PD-1 blockade did not affect iSTIP1 in lymphocytes, although 1 patient showed increased iSTIP1 in NK cells (**Figures 5G, H**). sSTIP1 was decreased in lymphocytes following anti-PD-1 treatment (**Figures 5G,H**). Since PD-1 blockade altered HSP90 expression in peripheral blood, we sought to determine whether similar changes occur in bone marrow lymphocytes. Consequently, we examined intracellular and extracellular HSP90β, TRAP1 and STIP1 in bone marrow lymphocytes from Patient 1 before and after the anti-PD-1 treatment and compared it to the HSP90 expression in peripheral blood of this patient (**Figure 6**). PD-1 blockade upregulated intracellular and downregulated surface HSP90β in BM-derived B cells (**Figures 6A, B**). By contrast, anti-PD-1 blockade downregulated intracellular and upregulated STIP1 expression in bone marrow B cells (**Figures 6E, F**). PD-1 blockade also downregulated intracellular and surface HSP90β and STIP1 expression in BM-derived NKT cells (**Figures 6A, B, E, F**). sHSP90β was decreased in peripheral blood and bone marrow B cells following anti-PD-1 therapy (**Figure 6B**). Interestingly, anti-PD-1 therapy resulted in decreased expression of iTRAP1 in BM-derived NK cells (**Figure 6C**). It is also interesting to note that PB- and BM- derived B cells differentially expressed iHSP90β after 24 hours of treatment with anti-PD-1 (**Figure 6A**). These findings suggest that anti-PD-1 treatment affects the frequency of lymphocytes and their intracellular and surface HSP90 expression in r/r cHL lymphoma patients, however, further studies are required to assess the effect of anti-PD-1 treatment on HSP90 expression and localization in bone marrow and peripheral blood lymphocytes.

### HSP90 Downregulation Affects NK Cell Degranulation Response and IFNγ Production in Healthy Donors and Lymphoma Patients

**Figure 7:**
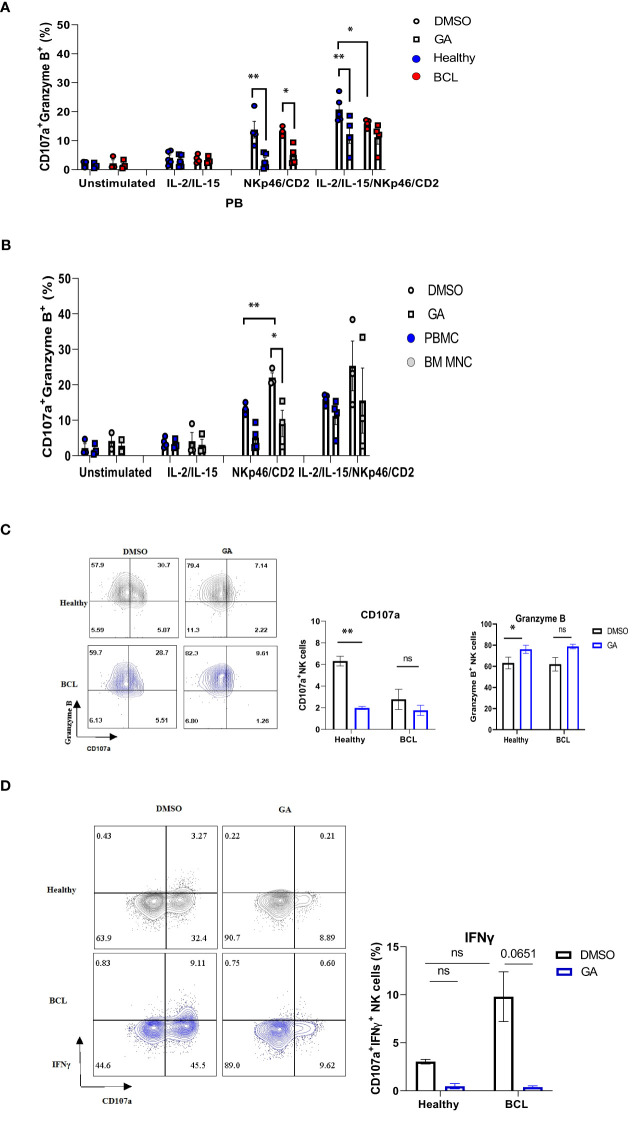
The effect of HSP90 inhibition on the NK cell degranulation response, granzyme B and IFNγ production in lymphoma patients. NK cell degranulation, as measured by CD107a surface expression, and granzyme B/IFNγ production in PB (n=4) of lymphoma patients and healthy controls (n=5) and BM (n=3) of lymphoma patients. PBMC **(A)** and BM MNC **(B)** were pre-treated with geldanamycin (0.1µM) or DMSO in the presence of IL-2 (100IU/ml) and IL-15 (10 ng/ml) and stimulated with anti-NKp46/anti-CD2. **(C)** Representative flow cytometry plots of CD107a^+^/Granzyme B^+^ double positive NK cells on the left and the frequency of CD107a single positive (SP) and Granzyme B SP NK cells in response to IL-2/IL-15 and anti-NKp46/anti-CD2 stimulation. **(D)** Representative flow cytometry plots of CD107a^+^ IFNγ^+^ NK cells on the left and the frequency of CD107a^+^IFNγ^+^ NK cells in response to IL-2/IL-15 and anti-NKp46/anti-CD2 stimulation. Graphs show mean ± SEM. ns, not significant, *p<0.05, **p< 0.01. GA, Geldanamycin; BCL, B-cell lymphoma.

Since anti-PD-1 treatment may alter HSP90 expression, we sought to determine whether modulating HSP90 level would affect the functional activity of NK cells. IL-2/IL-15-preactivated NK cells from healthy controls were more responsive to anti-NKp46/anti-CD2 stimulation, resulting in higher frequency of CD107a^+^ Granzyme B^+^ NK cells, as compared to NK cells from BCL patients (**Figure 7A**). By contrast, IL-2/IL-15/anti-NKp46/anti-CD2 stimulation resulted in increased frequency of CD107a^+^IFNγ^+^ NK cells in BCL patients, as compared to healthy controls (**Figure 7D**). HSP90 inhibition decreased the frequency of CD107a+/Granzyme B+ in healthy controls, bone marrow and peripheral blood NK cells of BCL patients (**Figures 7A, B**). HSP90 inhibition downregulated the expression of CD107a on the surface of bone marrow and peripheral blood NK cells, leading to double positive CD107a+ Granzyme B+ NK cells lose their CD107a+ expression and become single positive Granzyme B^+^ NK cells upon stimulation (**Figure 7C**). HSP90 inhibition also decreased the percentage of CD107^+^IFNγ^+^ NK cells in response to IL-2/IL-15 and anti-NKp46/anti-CD2 stimulation (**Figure 7D**). These data suggest that downregulating HSP90 may impair NK cell degranulation response and IFNγ production in lymphoma patients.

## Discussion

We have assessed the expression of constitutive and mitochondrial HSP90 and HSP90 co-chaperone STIP1/HOP in two major types of lymphoma- Hodgkin lymphoma and Non-Hodgkin lymphoma. We showed that two lymphomas differ by the expression of intracellular and surface content of HSP90β and STIP1 in peripheral blood and bone marrow lymphocytes. Intriguingly, peripheral blood B cells showed to be the major type of lymphocytes with abnormal expression of HSP90s inside and on their surface in lymphoma patients. HSP90β and STIP1 were also aberrantly expressed on the surface of bone marrow B cells in lymphoma patients. Since HSP90 overexpression may potentially be associated with circulating malignant B cell clones, we have performed B-cell clonality analysis. No malignant B cell clones were found in the blood and bone marrow of HL and NHL patients, suggesting that high HSP90 expression in B cells may not be associated with malignant phenotype. Several studies reported that extracellular HSPs associate with B regulatory phenotype (22, 23). In a recent study, Wang and colleagues reported that regulatory B cells have high expression of HSP70 (24). Along this line, Tang et al. demonstrated that extracellular BiP/GRP78 induces regulatory B cell phenotype (22). Extracellular HSP60 stimulates B cells to produce IL-10 and IL-6 while HSP60-stimulated B cells induce the proliferation and IFNγ and IL-10 production in T cells (23). These data suggest that altered expression of HSP90 in B cells may affect B cell responses in bone marrow and peripheral blood of lymphoma patients.

Recently, Zavareh and colleagues demonstrated that HSP90 inhibitors downregulate surface PD-L1 expression in mouse models *via* the regulation of HSP90 clients (c-Myc and STAT3) (25). HSP90 inhibition also showed to potentiate anti-tumor activity of PD-1 and CTLA-4 blockade *in vivo* (26, 27). Here, we showed that treatment with anti-PD-1 altered the expression and localization of HSP90β, TRAP1 and STIP1 in peripheral blood lymphocytes in refractory HL patients. PD-1 blockade also affected HSP90 content and localization in bone marrow lymphocytes. Notably, PD-1 blockade resulted in increased surface HSP90 expression in lymphocytes. Previous studies demonstrated that HSPs can be upregulated on the surface of immune cells following ER stress (28, 29). These studies suggest that there is interplay between immune checkpoints and HSP90s and that lymphocytes may upregulate surface HSP90 expression in response to anti-PD-1 immunotherapy, however, further studies are required to understand the role of cancer immunotherapy on the HSP90 expression.

Several studies reported high expression of PD-1 on NK cells in cancer patients (30, 31). Furthermore, Vari and colleagues highlighted an important role of PD-1/PD-L1 axis in the functional activity of NK cells in lymphoma patients (30). Taking into account that anti-PD-1 immunotherapy may affect HSP90 expression in NK cells, we assessed the effect of HSP90 downregulation on the degranulation response, granzyme B and IFNγ production in NK cells of lymphoma patients. We found that HSP90 inhibition downregulates CD107a expression and IFNγ production in NK cells upon stimulation. These results are consistent with previous findings showing that HSP90 inhibitors downregulate IFNγ secretion by NK cells (32). It is important to note that geldanamycin blocks HSP90 ATPase activity and thus, inhibits all four HSP90 isoforms, including HSP90α, HSP90β, TRAP1 and GRP94 in NK cells, suggesting that it is critical to identify specific HSP90 homolog responsible for the regulation of CD107a+ expression and IFNγ production in NK cells. Recent studies have demonstrated that cell metabolism plays a critical role in NK cell functional activity (33). Wang and colleagues reported that inhibition of glycolysis downregulates NK cell IFNγ production and CD107a expression (34). Authors also showed that glycolysis inhibition abrogated Granzyme B production while inhibition of oxidative phosphorylation (OXPHOS) did not affect Granzyme B production by NK cells (34). In our study we showed that geldanamycin inhibited CD107a expression and IFNγ production while Granzyme B production was not affected, suggesting that HSP90 inhibitor may affect both, i.e. glycolysis and OXPHOS. In this regard, mitochondrial HSP90 homolog TRAP1 showed to be a critical regulator of OXPHOS and glycolysis, suggesting that TRAP1 may be a potential isoform responsible for the downregulation of CD107a expression and IFNγ production in NK cells, however, this warrants further investigation (35, 36).

In summary, we showed that lymphoma patients have abnormal expression of HSP90s in bone marrow and peripheral blood B cells. PD-1 blockade altered the intracellular and surface HSP90 expression in immune population in r/r HL patients. Altering the level of HSP90 may inhibit cytotoxic activity of peripheral blood and bone marrow NK cells. Further understanding the effect of cancer immunotherapy on intracellular and extracellular HSP90 may help in identification of patients who will likely benefit from the treatment.

## Conclusion

HSP90 molecular chaperones play critical role in proteome homeostasis and showed to be implicated in various hallmarks of cancer. We show that constitutive, mitochondrial HSP90s and HSP90 co-chaperone STIP1/HOP are aberrantly expressed in B cells of lymphoma patients. Since approved and emerging cancer immunotherapeutics include immune checkpoint inhibitors, we have assessed the effect of anti-PD-1 treatment on HSP90 expression in refractory/relapsed lymphoma patients. We showed that anti-PD-1 affects HSP90 level and localization in immune cells of lymphoma patients. Additionally, we found that modulating HSP90 level may impair functional activity of NK cells. Further understanding of the effect of immunotherapies on HSP90 may improve treatment response in lymphoma patients.

## Data Availability Statement

The original contributions presented in the study are included in the article/**Supplementary Material**. Further inquiries can be directed to the corresponding author.

## Ethics Statement

The studies involving human participants were reviewed and approved by Research Ethics Committee of the Federal State Budgetary Institution ‘National Medical Research Center for Hematology’ of the Ministry of Health of the Russian Federation. The patients/participants provided their written informed consent to participate in this study.

## Author Contributions

ZA, conceived, designed and conducted experiments, analyzed the data, and wrote the manuscript. YM, provided clinical expertise and supervised clinical part of the project. AA, contributed to editing of the manuscript and funding acquisition. EN, contributed to the gene rearrangement analysis. SK, contributed to the clinical part of the project. AS, provided administrational support. All authors contributed to the article and approved the submitted version.

## Funding

This work was funded by RFBR, project number 20-315-90081. YM was supported by the RAKFOND grant 2020-02.

## Conflict of Interest

The authors declare that the research was conducted in the absence of any commercial or financial relationships that could be construed as a potential conflict of interest.

## Publisher’s Note

All claims expressed in this article are solely those of the authors and do not necessarily represent those of their affiliated organizations, or those of the publisher, the editors and the reviewers. Any product that may be evaluated in this article, or claim that may be made by its manufacturer, is not guaranteed or endorsed by the publisher.
