# Supplementary material for: Aberrant HSP90 Expression in Lymphocytes and HSP90 Response to Anti-PD-1 Therapy in Lymphoma Patients
Source: Front Immunol. 2022 Apr 28;13:893137. doi: 10.3389/fimmu.2022.893137 (PMC9095953; doi:10.3389/fimmu.2022.893137)
Supplement: Supplementary file 1 [file DataSheet_1.docx]

Supplementary Material

## Supplementary Figures

**
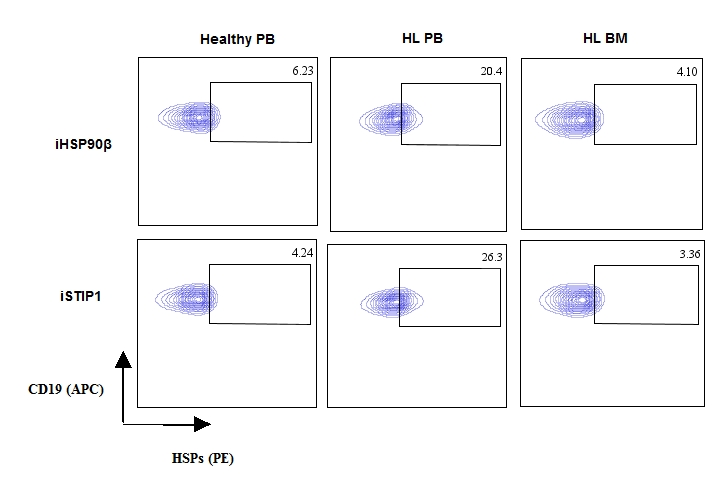
Supplementary Figure 1.** Representative plots of iHSP90β and iSTIP1 expression in PB and BM-derived B cells in HL patients. PB, peripheral blood; BM, bone marrow.


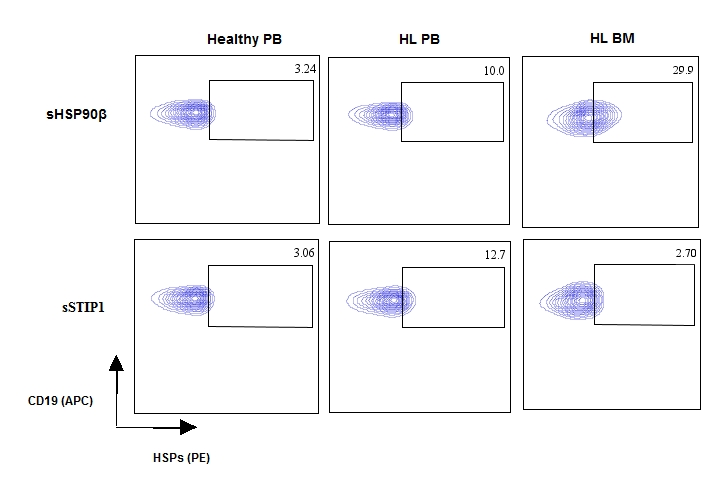


**Supplementary Figure 2.** Representative plots of sHSP90β and sSTIP1 expression in PB and BM-derived B cell in HL patients. PB, peripheral blood; BM, bone marrow.

**HL BM**

**
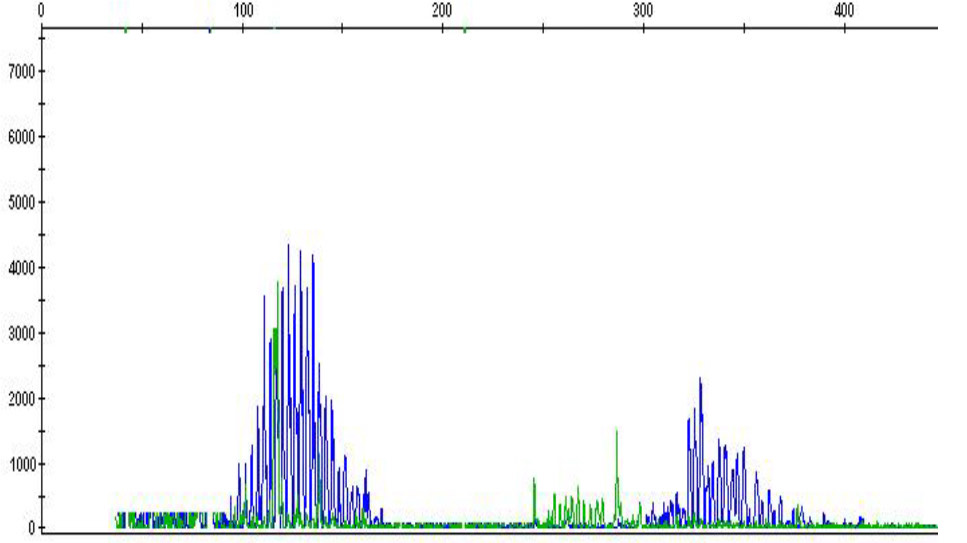
**

**NHL BM**

**
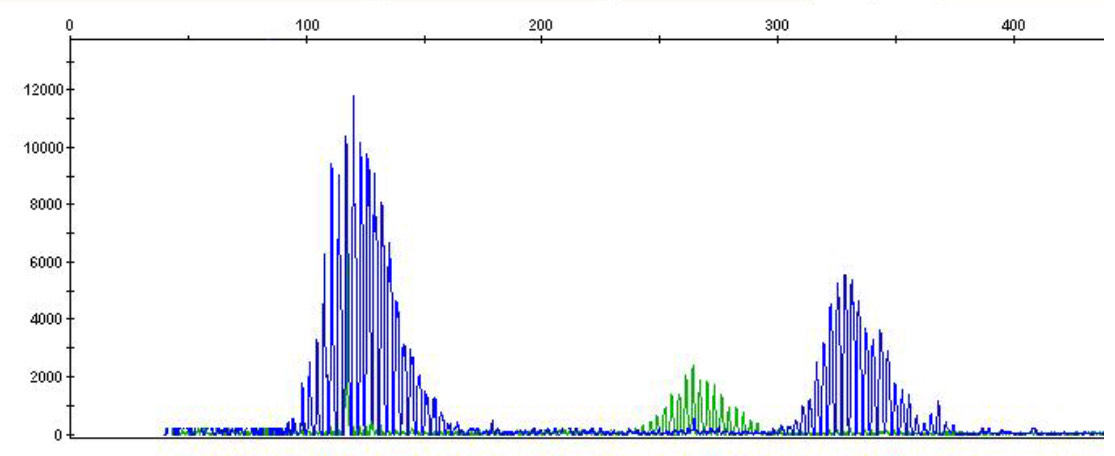
**

**NHL BM**

**
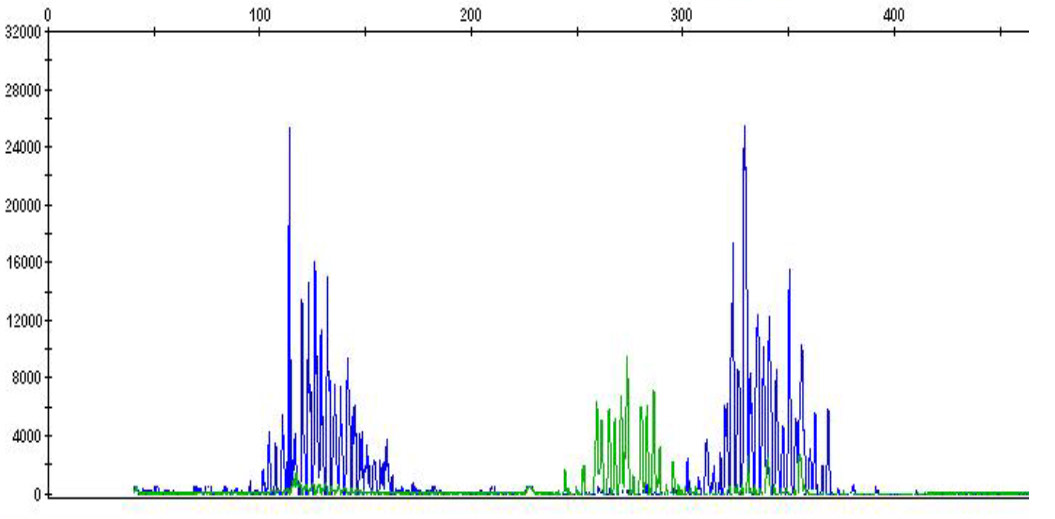
**

**NHL PB**

**
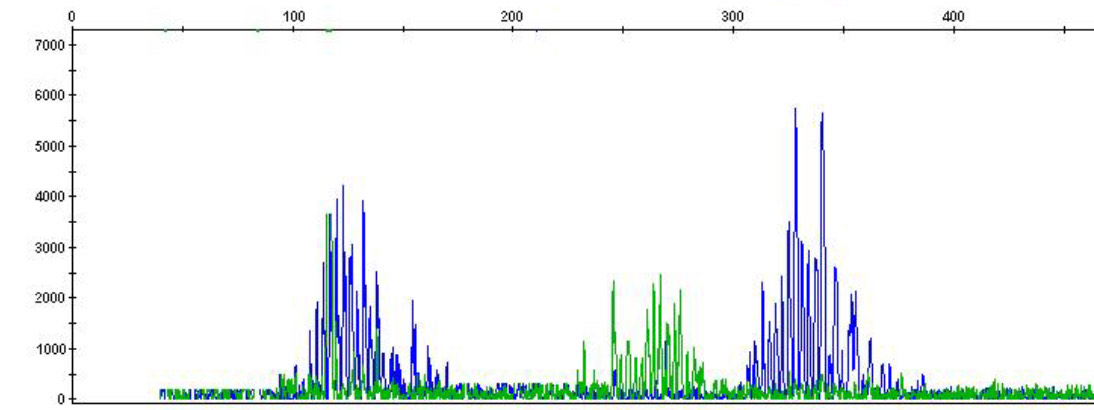
**

**Supplementary Figure 3.** B-cell clonality analysis in PB and BM of HL and NHL patients.
